# Determinants of Anemia among HIV-Positive Children on Highly Active Antiretroviral Therapy Attending Hospitals of North Wollo Zone, Amhara Region, Ethiopia, 2019: A Case-Control Study

**DOI:** 10.1155/2020/3720572

**Published:** 2020-02-18

**Authors:** Biruk Beletew, Ayelign Mengesha, Mohammed Ahmed, Awet Fitwi, Mesfin Wudu

**Affiliations:** ^1^Woldia University, Faculty of Health Sciences, Department of Nursing, P.O.Box 400, Woldia, Ethiopia; ^2^Woldia University, Faculty of Health Sciences, Department of Public Health, P.O.Box 400, Woldia, Ethiopia

## Abstract

*Introduction*. Anemia is one of the most commonly observed hematological abnormalities and an independent poor prognostic marker of HIV disease. The rate of progression and mortality in this subgroup of patients is high compared to nonanemic patients. WHO estimates that over two billion people are anemic worldwide and young children bear the world's highest prevalence rate of anemia. In Ethiopia, there is limited information about the determinant factors associated with anemia among HIV positive children. Thus, this study aimed to determine the determinant factors of anemia among HIV-infected children on HAART.

**Objective:**

The main purpose of this study was to assess the determinants of anemia among children on highly active antiretroviral therapy attending hospitals of North Wollo Zone, Amhara Region, Ethiopia.

**Methods:**

A case-control study was conducted on 350 HIV-infected children on HAART attending Hospitals of North Wollo Zone, from February 1 to March 30, 2019. The study participants were selected with a consecutive sampling technique. An adapted, interviewer-administered, and pretested questionnaire and chart review were employed to collect the data. Besides, blood and stool samples were investigated to determine hematologic indices and malaria and to investigate intestinal parasites, respectively. Data were analyzed by using the SPSS version 24 statistical software and bivariate and multivariate logistic regression was used to identify predictors.

**Results:**

A total of 350 HIV positive children (117 cases and 234 controls) were included in this study with an overall response rate of 100%. On multivariate analysis, variables which have spastically significant association with anemia were as follows: had amebiasis (AOR = 7.29, 1.22–43.56), had history of opportunistic infections (AOR = 9.63, 1.94–47.85), had malaria infection (malaria pf) (AOR = 4.37, 1.16–16.42), eating nondiversified food (AOR = 10.39, 2.25–48.0), WGT-Age *Z* score value between −2_−3 (AOR = 9.80, 2.46–39.14), level of adherence (AOR = 2.31, 1.92, 7.77), and being from a rural area (AOR = 8.8, 2.07–37.79).

**Conclusion:**

In this study, having parasitic infections, having a history of opportunistic infections, being malnourished, having poor adherence to ART, caregivers living in the rural area, and eating nondiversified foods were significantly associated with hemoglobin status. Therefore, intervention aimed at prevention, early diagnosis, and treatment of anemia is essential in these patients.

## 1. Background

Hematological abnormalities are a common occurrence in individuals diagnosed with human immunodeficiency virus (HIV) [1–5]. Of these hematological abnormalities, anemia is the most common in HIV-infected children and it has a significant impact on the quality of life and clinical outcomes [3–5].

According to World Health Organization (WHO) definitions, anemia is defined as having a hemoglobin level less than 11 g/dl for children <5 years old, <11.5 g/dl for children 5–11.9 years old, and <12 g/dl for children 12–14.9 years old after altitude adjustment [6]. Anemia is classified as mild (Hgb 10.0–10.9 g/dl), moderate (Hgb 7.0–9.9 g/dl), severe (Hgb 4.0–7.0 g/dl), and very severe (Hgb less than 4.0 g/dl). It can also be classified based on the hematocrit (PCV) percent. Packed cell volume (PCV) of less than 33.0% is regarded as anemia by the World Health Organization [6].

The risk factors of anemia are multiple and vary across geographical areas. In HIV-infected patients, anemia may be caused by nutrient deficiencies (iron, folic acid, and vitamin B12), sickle cell disease, HIV/AIDS itself, malaria, hookworm, and other infections. Iron deficiency anemia is the leading (50%) cause of childhood anemia in developing countries [4, 5, 7].

In addition to the direct effect of HIV, highly active antiretroviral therapy (HAART) also causes anemia. Although HAART has the capability of reducing the incidence of anemia [8] by suppressing viral replication and increases CD4 cell count, anemia remains a common problem even for patients treated with antiretroviral agents. Anemia due to drugs, such as cotrimoxazole, pentamidine, foscarnet, and zidovudine (AZT), often reflects reticuloendothelial iron block [9].

There are several factors believed to contribute to the pathophysiology of anemia observed in HIV patients. First, many of the opportunistic infections or malignancies to which HIV patients are susceptible can lead to anemia [10].

As with anemia in the HIV population, micronutrient deficiencies can play a role in contributing to anemia in HIV patients [10]. Finally, many of the most common ART drugs included in the standard also harm hematopoiesis and can thus contribute to anemia in HIV. The molecular mimicry between erythropoietin (EPO) and HIV-1 p17 protein can lead to circulating auto-antibodies against endogenous EPO in some HIV patients [10, 11].

Anemia is a common comorbid condition among HIV-infected children and has a profound impact on disease progression and has been noted as a significant predictor of decreased survival time and death [12, 13]. Especially, children are more prone to the consequences of anemia due to high iron requirements, low intake of iron from foods, and frequent episodes of infection [6].

Anemia is one of the global public health problems that affect more than one third of the world population. It has been strongly associated with poor growth and development, limited psychomotor development, and poor long-term performance in cognitive, social, and emotional functioning in children. It is also associated with impaired mental, physical, and language development and poor coordination, scholastic achievement, and immune function [14].

Anemia is a common childhood health problem in Africa, and its prevalence among children under the age of five years is estimated at 62%, which is above the cut-off points (40%). It is a leading cause of pediatric mortality and impaired development and is highly prevalent in young children in sub-Saharan Africa [5, 15].

In Ethiopia, as many as six in ten under-five children are anemic [16]. As per EDHS 2016, overall, more than half of the children 6–59 months (56 percent) suffered from some degree of anemia: 25 percent were mildly anemic, 28 percent were moderately anemic, and 3 percent were severely anemic [7]. Despite causing such high levels of burden, the public health community does not pay anemia the attention it deserves.

The government of Ethiopia has been working to reduce childhood anemia. Accordingly, it endorsed the national nutrition program and bimanual school deworming, developed micronutrient deficiency prevention and control guideline, and implemented micronutrient fortification. But studies from different corners of the country have shown that childhood anemia is still a major public health problem in this country [17].

Most of the studies on the HIV-anemia interplay were done in developed countries and mainly included adults. Determinants of anemia among HIV/AIDs positive children on HAART have not been very well understood in resource-poor settings like Ethiopia, although there are few cross-sectional studies conducted at different parts of the country. However, knowing the determinants of anemia among HIV/AIDS positive children on HAART through the case-control study will help to screen and manage anemia according to its cause. Therefore, the main aim of this study is to assess the determinant factors of anemia among HIV-positive children on highly active antiretroviral therapy (HAART) attending hospitals of North Wollo Zone, Amhara, Ethiopia, 2019.

## 2. Methodology

### 2.1. Study Area

This study was carried out in North Wollo Zone Hospitals, Amhara Region, Ethiopia. According to the 2007 Census conducted by the Central Statistical Agency of Ethiopia (CSA), this zone has a total population of 1,500,303, of whom 752,895 are men and 747,408 women. The largest ethnic group, language spoken, and religion followed in this area is Amhara (99.38%), Amharic language (99.28%), and Ethiopian Orthodox Christianity (82.74%), respectively. There are five public hospitals in this zone.

### 2.2. Study Design and Period

A health facility-based unmatched case-control study was conducted from February 1 to March 30, 2019.

#### 2.2.1. Study Population

All HIV-positive children on highly active antiretroviral therapy (HAART) attended the selected hospitals of North Wollo Zone, Amhara, Ethiopia.

### 2.3. Sample Size Determination

The sample size is determined using a double population proportion difference formula using iron deficiency as an independent predictor (exposure) variable since it gives the maximum sample size. From that study, the proportion of children among cases with iron deficiency was 26.9% and the proportion of children among controls with iron deficiency was 8.3% [18]. Using Epi-Info version 7, one to two ratio (1 : 2) of cases to controls, and a power of 90%, the total sample size becomes 350 with 117 cases and 234 controls.

## 3. Sampling Technique and Procedure

Simple random sampling technique was employed to select the required number of cases and controls attending hospitals in North Wollo Zone. First, out of the total five hospitals in North Wollo Zone, three hospitals were selected through simple random sampling technique. Then, the required sample size was allocated proportionally to the selected hospitals based on the number of cases and controls. Study participants (cases and controls) in the selected hospitals were selected using consecutive sampling technique by considering that the client flow is random. Cases were selected consecutively among HIV-positive children on HAART in the selected hospitals. The next immediate two corresponding age- and sex-matched controls were selected consecutively among HIV-positive children on HAART in the selected hospitals.

(i) Variables
  (1) Dependent variables
    - Anemia (yes/no)
(ii) Independent variables
  (1) Sociodemographic variables
    - Ethnicity
    - Residence
    - Religion
    - Occupation of mother
    - Family size
    - Household wealth index status
    - Altitude
    - The educational level of the mother
    - The educational level of the father
    - Age
    - Sex
  (2) Disease-related
    - Cd4 count/percentage
    - WHO stage
    - Stool result (IP)
    - Malaria history
    - Opportunistic infection (OPs)
    - Inflammation (ultrasensitive CRP > 1.0 mg/dl)
    - Past medical history
  (3) Drug-related
    - HAART duration
    - HAART status (new….experienced…)
    - HAART regimen
    - Cotrimoxazole
    - Previous and current drug history
  (4) Nutritional related variables
    - Dietary Diversity Score
    - Nutritional status
(iii) Eligibility criteria
  (1) Inclusion criteria
    - Inclusion criteria for cases. HIV-positive children on highly active antiretroviral therapy (HAART) who were anemic and may not be diagnosed with other medical illness.
    - Inclusion criteria for controls. HIV-positive children on highly active antiretroviral therapy (HAART) who were not anemic
    - Exclusion criteria for cases and controls. Children with an incomplete chart, children who passed away on arrival, and children who were admitted without their caregiver.

### 3.1. Definitions of Terms and Operational Definitions

- Case (anemic): HIV-positive children on Highly Active Antiretroviral Therapy (HAART) whose Hg level is less 11 g/dl for children <5 years old, <11.5 g/dl for children 5–11.9 years old, and <12 g/dl for children 12–14.9 years old or who may not be diagnosed with other medical illness [19].
- Control (nonanemic): HIV-positive children on Highly Active Antiretroviral Therapy (HAART) whose Hg level is more than or equal to 11 g/dl for children <5 years old, 11.5 g/dl for children 5–11.9 years old, and 12 g/dl for children 12–14.9 years old and they are age- and sex-matched.
- Mild anemia (Hgb 10.0–10.9 g/dl), moderate anemia (Hgb 7.0–9.9 g/dl), severe anemia (Hb < 7.0 g/dl for children aged 6–59 months and Hb < 8.0 for those aged 5 years and older) [20, 21].
- Very severe anemia (Hgb less than 4.0 g/dl).

### 3.2. Data Collection Tool and Procedure

Data was collected by face-to-face interview, chart review, and anthropometric measurement with pretested and structured questionnaire adapted from previous similar literature [13, 14]. A review of the medical records was performed for concurrent opportunistic infections and WHO clinical staging of HIV disease. Laboratory investigation was performed by laboratory professionals to determine the hemoglobin level of participants and identify possible malaria and other parasitic infections [18]. The questionnaire had five parts: Part 1, sociodemographic/economic variables; part 2, assessment of degree and type of anemia; part 3: disease-related determinants of anemia; part 4, drug-related determinants of anemia; part 5, nutritional status-related determinants of anemia.

- Nutritional assessment: Dietary Diversity Score (DDS) was calculated from single 24-hour dietary recall data. Additionally, study participants were screened for nutritional status using anthropometric measurements.
- Chart review was done to identify hemoglobin concentration (Hgb), blood history of malaria, and viral load.
- Stool specimen collection and examination: a stool sample was collected from each study participants using clean, wide-mouthed, and leak-proof stool cup by laboratory professionals. Then, stool wet mount was prepared using saline and/or iodine and examined microscopically for identification of intestinal helminths.

### 3.3. Data Quality Control

A pretest was conducted in Dessie Referral Hospital on 5% [8] of the sample size. Training was given for data collectors and supervisors. On-spot checking and correction were made for incomplete questionnaires. Laboratory test quality was assured by giving training for laboratory professionals by MSc laboratory professionals using the standard operating procedure (SOP) and regular monitoring of reagents for manufacturing, expiry date, and proper storage. The sample was processed immediately after collecting from the study participants to minimize errors.

### 3.4. Data Analysis and Presentation

The filled questionnaires were checked for completeness, coded, and entered into Epi Data version 4.2 and then exported to SPSS version 24 for further analysis. Descriptive statistics were computed and the result was reported using frequencies and percentages. Bivariate logistic regression was performed and variables with *P* < 0.25 were transported to multivariable logistic regression. Finally, variables with *P* value <0.05 in the multivariable logistic regression model were taken as statistically significant and the adjusted odds ratio with its 95% confidence interval was considered to see the strength of association between the exposure variables and the outcome of interest.

### 3.5. Ethical Consideration

Ethical clearance was obtained from the Woldia University Research Project Office. Permission and support letter was sought and obtained from the North Wollo Zone Health Office. Then, officials at different levels in the hospitals were communicated through letters. The responsible bodies at each hospital were told about the purpose of the study. Written informed consent was obtained from parents of every study participants after a detailed explanation on the purpose and benefit of the study right before data collection and assent was obtained from the study participants. All data collectors and supervisors were instructed on how to comply with strict confidentiality practices for all participants both during and after data collection. The identity of the participants was kept anonymous.

### 3.6. Dissemination of the Result

The results of the study were disseminated to Woldia University, faculty of health science, research directorate office. Furthermore, the findings and recommendations of the study will also be shared in a written document to North Wollo Zone Health Office and Hospitals in North Wollo Zone. After public defense and incorporation of comments, the results will also be disseminated through presentations on research conferences, workshops, and symposiums. Finally, an attempt will be made to publish the results in a reputable peer-reviewed journal. The study participants will remain anonymous during and after publication. The owners of the research are the researchers and Woldia University.

## 4. Results

### 4.1. Sociodemographic Characteristics of the Respondents

A total of 350 HIV positive children (117 cases and 234 controls) were included in this study with an overall response rate of 100%. One hundred eighty two 182 (52%) of them were females. The median age of the child was 3.14 years. Most of the participants were from urban areas (24.2%% cases and 34.9% controls). Regarding religion, 206 (58.9%) were Orthodox Christians followed by Muslims, 110 (31.4%). Regarding the marital status of mothers, 72 (20.6%) cases and 136 (38.9%) controls were married. Regarding the educational status of mothers, 59 (16.9%) cases and 85 (24.3%) controls were illiterate and the remaining were able to read and write or had completed primary education and above. Regarding occupation of mothers, the majority of them 66 (18.9%) cases and 83 (23.7%) controls were nonemployed and 44 (12.6%) cases and 83 (23.7%) controls were housewives. From the total study participants, 42 (12.0%) cases and 146 (41.7%) controls were from middle-income class families (Table 1).

### 4.2. Nutrition, Disease, and Drug-Related Predictors of Anemia among Children on HAART

This study revealed that 36.6% of children on HAART, 62 (17.7%) cases and 66 (18.9%) controls, had a history of severe acute malnutrition. Similarly, 86 (24.6%) cases and 114 (32.6%) controls had a history of opportunistic infections. The proportion of malaria infection was around two times higher among cases, 58 (16.6%), compared to controls, 31 (8.9%). Besides, the proportion of having low CD4-count (<350) was approximately sixfold higher among cases, 56 (16.0%), compared to controls, 10 (2.9%). Regarding HAART, the proportion of poor adherence was around three times higher among cases, 16 (4.6%), compared to controls, 5 (1.4%) (Table 2).

### 4.3. Regression Analysis on Factors Associated with Anemia among Children on HAART

Bivariate logistic regression analysis was done for different predictor variables. Intestinal parasite, WGT-AGE *Z* score, history of oropharyngeal disease, history of opportunistic infections, history of chronic diarrhea, history of missing follow-up, HAART duration, level of adherence to HAART, being on cotrimoxazole, viral load, malaria history, highest WHO staging, caregiver's residence, orphanhood of the child, wealth index, household food security, food diversity, feeding frequency, and counselling on child feeding were significantly associated with the HGB level of respondents. All variables with a value less than 0.25 were fitted into the backward stepwise multivariate logistic regression model. But, on multivariate analysis, having parasitic infections, a history of opportunistic infections, being malnourished, poor adherence to ART, caregivers living in the rural area, and eating nondiversified foods were significantly associated with anemia among HIV positive children on HAART.

The odds of being anemic among HIV-positive children on HAART who had amebiasis was around seven times higher than those who had no amebiasis (AOR = 7.29, 1.22–43.56). Similarly, the odds of children on HAART who had a history of opportunistic infections was about 10 times higher to be anemic compared to those who had no history of opportunistic infections (AOR = 9.63, 1.94–47.85). The odds of being anemic among children on HAART who had malaria infection (malaria pf) was also around fourfold higher than those who have no malaria infection (AOR = 4.37, 1.16–16.42).

Eating nondiversified foods was found to be significantly associated with anemia among children on HAART. The odds of being anemic among HIV positive children on HAART who eat nondiversified foods was about tenfold higher than those who eat diversified foods (AOR = 10.39, 2.25–48.0). At the same time, the odds of children on HAART with WGT–Age *Z* score value between −2_−3 was also about 10 times higher to be anemic than those with WGT–Age *Z* score value between 1_−2 (AOR = 9.80, 2.46–39.14).

The odds of children on HAART who have poor adherence to ART drugs were two times higher compared to those who have good adherence (AOR = 2.31, 1.92, 7.77). Besides, caregivers residence was also significantly associated with anemia. The odds of being anemic among children on HAART whose caregiver lives in the rural area was approximately ninefold higher than those with a caregiver living in an urban area (AOR = 8.8, 2.07–37.79) (Table 3).

## 5. Discussion

Anemia is a common comorbid condition among HIV-infected children and has a profound impact on disease progression and has been noted as a significant predictor of decreased survival time and death [12, 13]. Especially, children are more prone to the consequences of anemia due to high iron requirements, low intake of iron from foods, and frequent episodes of infection [6].

This case-control study included sociodemographic variables like residence, occupation of mothers, household wealth index status, and disease-related variables like history malaria and opportunistic infections. It also included drug-related and nutrition-related variables like HAART duration, HAART regimen, dietary diversity score, and nutritional status of children. This study result revealed that the odds of being anemic among HIV-positive children on HAART who had amebiasis was around 7 times higher than those who had no amebiasis. Similarly, research from Addis Ababa, Ethiopia, revealed that having intestinal parasitic infections (AOR = 2.7, 95% CI, 1.1–7.2) was identified as a significant predictor of anemia [22]. This might be because some parasitic infections including malaria are the major causes of blood loss and destruction of red blood cells and also because amebiasis causes dysentery which will result in GI blood loss and in turn anemia. This implies that screening and treatment of intestinal parasites such as amebiasis can prevent anemia among HIV positive children [23].

Similarly, the odds of being anemic among children on HAART who had malaria infection (malaria pf) were around fourfold higher than those who have no malaria infection. This finding is in line with a study conducted at Zewditu Memorial Hospital, Ethiopia, in which intestinal parasites and malaria were independently associated with the odds of being anemic [17]. It is also consistent with research in South-Central Ethiopia in that the risk of anemia was high (adjusted hazard ratio = 10) among children with malaria [24]. The possible justification for this might be because falcifarum malaria can cause hemolysis which in turn results in hemolytic anemia. This shows the need to target interventions at HIV-positive children in prevention of malaria because the groups are at the highest risk of anemia due to malaria [25].

The odds of children on HAART who had a history of opportunistic infections were about 10 times higher to be anemic compared to those who had no history of opportunistic infections. This is consistent with a study at Zewditu Memorial Hospital in which TB-HIV coinfection was independently associated with the odds of being anemic [17]. This could be because children who are attacked by the opportunistic infection will have a loss of appetite and are more likely to be malnourished, which is a significant predictor of anemia [26]. This implies that the prevention of opportunistic infections is one of the important strategies to prevent anemia in HIV-positive children.

Eating nondiversified foods was found to be significantly associated with anemia among children on HAART. This is consistent with a research finding from Waghimra, Northeast Ethiopia, which revealed poor dietary diversity being significantly associated with anemia [27]. This is because when children receive diversified food, they will have access to iron-rich foods which will prevent iron deficiency anemia.

The odds of children on HAART with WGT–Age *Z* score value between −2_−3 was also about 10 times higher to be anemic than those with WGT-Age *Z* score value between −1_−2. This is in line with findings of other studies in which stunting and vitamin A deficiency were identified as an independent risk factor for anemia [27, 28]. This might be because malnourished children will have associated iron deficiency which is the most common cause of anemia.

The odds of children on HAART who have poor adherence to ART drugs were two times higher compared to those who have good adherence. Similar findings have been reported in other studies [29, 30, 31]. The possible justification for this might be that those children with poor adherence are at a higher risk of developing opportunistic infections which in turn results in anemia.

Besides, caregiver's residence was also significantly associated with anemia. The odds of being anemic among children on HAART whose caregiver lives in a rural area were approximately nine fold higher than those with a caregiver living in an urban area. This is in line with other reports in which children in rural areas were more likely to be anemic compared to those in urban areas [32, 33, 34]. This might be because families of children living in urban areas consume diversified foods in a better way; in addition to this, the prevalence of intestinal parasites is low in urban areas. Interventions to improve dietary diversification accompanied by opportunistic diseases control including malaria and intestinal parasites are needed.

## 6. Conclusion

In this study, having parasitic infections, a history of opportunistic infections, being malnourished, having poor adherence to ART, having caregivers living in the rural area, and eating nondiversified foods were significantly associated with anemia among HIV-positive children on HAART. This implies that implementation of an integrated package of interventions is essential to design an effective, context-specific anemia prevention and control program.

## Figures and Tables

**Table 1:** Sociodemographic characteristics of children on HAART and their parents in North Wollo Zone public hospitals, Amhara region, Ethiopia, 2019 (*N* = 350). Nutrition, disease, and drug-related predictors of anemia among children on HAART.

Exposure variable	Responses	Anemia status	
		Anemic count (%)	Nonanemic count (%)
Ethnicity	Amhara	113 (32.3)	217 (62.0)
	Oromo	1 (0.3)	5 (1.4)
	Tigrie	3 (0.9)	11 (3.1)

Educational status of the mother	Cannot read and write	59 (16.9)	85 (24.3)
	Can read and write	45 (12.9)	80 (22.9)
	Primary	12 (3.4)	59 (16.9)
	Secondary and above	1 (0.3)	9 (2.6)

Educational status of the father	Cannot read and write	53 (15.1)	89 (25.4)
	Can read and write	51 (14.6)	58 (16.6)
	Primary	6 (1.7)	63 (18.0)
	Secondary and above	7 (2.0)	23 (6.5)

Occupation of the mother	Not employed	66 (18.9)	83 (23.7)
	Housewife	44 (12.6)	83 (23.7)
	Governmental employee	6 (1.7)	62 (17.7)
	An employee in the private sector	1 (0.3)	5 (1.4)

Religion	Orthodox	72 (20.6)	134 (38.3)
	Muslim	31 (8.9)	79 (22.6)
	Others	14 (4)	20 (5.7)

Marital status of the mother	Single	15 (4.3)	16 (4.6)
	Married	72 (20.6)	136 (38.9)
	Divorced/Separated	30 (8.6)	74 (21.2)

Caregiver's educational status	Noneducated	55 (15.9)	74 (21.3)
	Primary	38 (11.0)	107 (30.8)
	Secondary or above	24 (6.9)	49 (14.1)

Caregiver's residence	Rural	33 (9.5)	109 (31.4)
	Urban	84 (24.2)	121 (34.9)

Caregiver being on ART	Yes	95 (27.1)	190 (54.3)
	No	22 (6.3)	43 (12.3)

The orphanhood of the child	Both	26 (7.4)	9 (2.6)
	Only mother	9 (2.6)	31 (8.9)
	Only father	4 (1.1)	15 (4.3)
	Not an orphan	78 (22.3)	178 (50.9)

Wealth index	1.00	43 (12.3)	30 (8.6)
	2.00	42 (12.0)	146 (41.7)
	3.00	32 (9.1)	57 (16.3)

Family size	<4	9 (2.6)	23 (6.6)
	4–6	49 (14.0)	123 (35.1)
	>6	59 (16.9)	87 (24.9)

Household food security	Not secured	23 (6.6)	33 (9.4)
	Secured	94 (26.9)	198 (56.6)

Sex of child	Male	67 (19.1)	101 (28.9)
	Female	50 (14.3)	132 (37.7)

Orphan status of the child	Yes	85 (24.3)	139 (39.7)
	No	32 (9.1)	55 (15.7)

Age of the child	1–5	69 (22.4)	108 (33.5)
	5–10	31 (10.1)	76 (24.7)
	10–18	17 (5.5)	46 (14.9)

**Table 2:** Description of exposure variable with their anemia status.

Exposure variable	Responses	Anemia status	
		Anemic count (%)	Nonanemic count (%)
Intestinal parasite	Negative	101 (28.9)	79 (22.6)
	*G. lamblia*	15 (4.3)	145 (41.4)
	*E. histolytica*	1 (0.3)	9 (2.6)
	*A. lumbricoides*	7 (1.2)	9 (1.6)
	Others	6 (1.1)	8 (1.5)

WGT-AGE *Z* score	−1_−2	44 (12.6)	166 (47.4)
	−2_−3	22 (6.3)	31 (8.9)
	<−3	51 (14.6)	36 (10.3)

Hx of oropharyngeal disease	Yes	37 (10.6)	46 (13.1)
	No	80 (22.9)	187 (53.4)

Hx of opportunistic infections	Yes	86 (24.6)	114 (32.6)
	No	31 (8.9)	119 (34.0)

Hx of chronic diarrhea	Yes	51 (14.6)	40 (11.4)
	No	66 (18.9)	193 (55.1)

Hx of missing follow-up	Yes	56 (16.0)	67 (19.1)
	No	61 (17.4)	166 (47.4)

HAART_duration	≤5 years	80 (22.9)	177 (50.6)
	>5 years	37 (10.6)	56 (16.0)

Level of adherence to HAART	Good	42 (12.0)	162 (46.3)
	Fair	59 (16.9)	66 (18.9)
	Poor	16 (4.6)	5 (1.4)

On cotrimoxazole	Yes	64 (18.3)	106 (30.3)
	No	53 (15.1)	127 (36.3)

Viral load	<1000	51 (14.6)	67 (19.1)
	>1000	66 (18.9)	166 (47.4)

Malaria history	Yes	58 (16.6)	31 (8.9)
	No	59 (16.9)	202 (57.7)

Highest WHO staging	Stage 4	21 (6.0)	33 (9.4)
	Stage 3	51 (14.6)	108 (30.9)
	Stage 2	32 (9.1)	67 (19.1)
	Stage 1	13 (3.7)	25 (7.1)

Household food security	Not secured	23 (6.6)	33 (9.4)
	Secured	94 (26.9)	200 (57.2)

Food diversity	Nondiversified	99 (28.3)	87 (24.9)
	Diversified	18 (5.1)	146 (41.7)

Feeding frequency	Not adequate	49 (14.0)	44 (12.6)
	Adequate	68 (19.4)	189 (54.0)

Counselling on child feeding	Yes	76 (21.7)	175 (50.0)
	No	41 (11.7)	58 (16.6)

**Table 3:** Regression analysis on predictors of anemia among children on HAART in North Wollo Zone Public Hospitals, Amhara region, Ethiopia, 2019 (*n* = 350).

Exposure variable	Responses	Anemia status		Bivariate and multivariate regression analysis		
		Anemic count (%)	Nonanemic count (%)	COR (95% CI)	AOR (95% CI)	*P*-value
Intestinal parasite	Negative	101 (28.9)	79 (22.6)	1	1	
	*G. lamblia*	15 (4.3)	145 (41.4)	9.09 (4.83, 17.11)	0.1 (0.02, 0.49)	0.005
	*E. histolytica*	1 (0.3)	9 (2.6)	9.54 (4.71, 19.35)	7.29 (1.22, 43.56)	**0.029**
	*A. lumbricoides*	7 (1.2)	9 (1.40)	7.19 (2.49, 20.69)	0.63 (0.06, 6.39)	0.694
	Others	6 (1.1)	8 (1.3)	7.46 (2.43, 22.94)	2.49 (0.27, 23.01)	0.422

WGT-AGE *Z* score	−1_−2	44 (12.6)	166 (47.4)	1	1	
	−2_−3	22 (6.3)	31 (8.9)	2.67 (1.41, 5.07)	9.80 (2.46, 39.14)	**0.001**
	<−3	51 (14.6)	36 (10.3)	5.35 (3.11, 9.18)	2.46 (0.63, 9.69)	0.198

Hx of oropharyngeal disease	Yes	37 (10.6)	46 (13.1)	1.88 (1.13, 3.12)	0.40 (0.10, 1.57)	0.187
	No	80 (22.9)	187 (53.4)	1	1	

Hx of opportunistic infections	Yes	86 (24.6)	114 (32.6)	1.88 (1.13, 3.12)	9.63 (1.94, 47.85)	**0.006**
	No	31 (8.9)	119 (34.0)	1	1	

Hx of chronic diarrhea	Yes	51 (14.6)	40 (11.4)	2.89 (1.78, 4.70)	1.05 (0.23, 4.87)	0.949
	No	66 (18.9)	193 (55.1)	1	1	

Hx of missing follow-up	Yes	56 (16.0)	67 (19.1)	3.73 (2.26, 6.14)	0.38 (0.09, 1.54)	0.174
	No	61 (17.4)	166 (47.4)	1	1	

HAART_duration	≤5 years	80 (22.9)	177 (50.6)	1.46 (0.89, 2.39)	2.33 (0.69, 7.94)	0.175
	>5 years	37 (10.6)	56 (16.0)	1	1	

Level of adherence to HAART	Good	72 (20.6)	132 (37.7)	1	1	
	Fair	59 (16.9)	66 (18.9)	1.64 (2.10, 5.56)	1.33 (0.69, 7.94)	0.175
	Poor	13 (3.7)	8 (2.3)	3.0 (4.18, 9.63)	2.31 (1.92, 7.77)	0.000

On cotrimoxazole	Yes	64 (18.3)	106 (30.3)	1.45 (93, 2.26)	0.33 (0.09 (1.14)	0.079
	No	53 (15.1)	127 (36.3)	1	1	

Viral load	<1000	51 (14.6)	67 (19.1)	1.92 (1.21, 3.04)	1.09 (0.23, 5.18)	0.92
	>1000	66 (18.9)	166 (47.4)	1	1	

Malaria history	Yes	58 (16.6)	31 (8.9)	6.41 (3.79, 10.82)	4.37 (1.16, 16.42)	**0.029**
	No	59 (16.9)	202 (57.7)	1	1	

Highest WHO staging	Stage 4	21 (6.0)	33 (9.4)	1.22 (0.52, 2.91)	0.01 (0.00, 0.16)	0.002
	Stage 3	51 (14.6)	108 (30.9)	0.91 (0.43, 1.919)	0.03 (0.00, 0.26)	0.002
	Stage 2	32 (9.1)	67 (19.1)	0.92 (0.42, 2.03)	0.022 (0.00, 0.23)	0.001
	Stage 1	13 (3.7)	25 (7.1)	1	1	

Caregiver's residence	Rural	33 (9.5)	109 (31.4)	2.28 (1.41, 3.67)	8.85 (2.07, 37.79)	**0.003**
	Urban	84 (24.2)	121 (34.9)	1	1	

The orphanhood of the child	Both	26 (7.4)	9 (2.6)	6.59 (2.95, 14.72)	7.92 (0.66, 94.77)	0.102
	Only mother	9 (2.6)	31 (8.9)	0.66 (0.30, 1.46)	0.74 (0.15, 3.50)	0.699
	Only father	4 (1.1)	15 (4.3)	0.61 (0.19, 1.89)	2.31 (0.17, 31.62)	0.529
	Non-orphan	78 (22.3)	178 (50.9)	1	1	

Wealth index	1.00	43 (12.3)	30 (8.6)	2.55 (1.35, 4.82)	0.64 (0.09, 4.84)	0.665
	2.00	42 (12.0)	146 (41.7)	0.51 (0.29, 0.89)	0.76 (0.17, 3.26)	0.707
	3.00	32 (9.1)	57 (16.3)	1	1	

Household food security	Not secured	23 (6.6)	33 (9.4)	1.48 (0.83, 2.66)	0.43 (0.07, 2.75)	0.370
	Secured	94 (26.9)	200 (57.2)	1	1	

Food diversity	Nondiversified	99 (28.3)	87 (24.9)	9.23 (5.23, 16.28)	10.39 (2.25, 48.0)	**0.003**
	Diversified	18 (5.1)	146 (41.7)	1	1	

Feeding frequency	Not adequate	49 (14.0)	44 (12.6)	9.23 (5.23, 16.29)	1.62 (0.35, 7.59)	0.539
	Adequate	68 (19.4)	189 (54.0)	1	1	

Counselling on child feeding	Yes	76 (21.7)	175 (50.0)	1	1	
	No	41 (11.7)	58 (16.6)	1.63 (1.01, 2.64)	1.13 (0.24, 5.33)	0.876

## Data Availability

The data used to support this study are available from the corresponding author upon reasonable request.

## Abbreviations

ART: Antiretroviral therapy
CD4: Cluster of differentiation
EDHS: Ethiopia demographic health survey
HAART: Highly active antiretroviral therapy
HAZ: Height-for-age *Z*-score
HIV/AIDS: Human immunodeficiency virus/acquired immune deficiency syndrome
SPSS: Statistical package for social science
WHO: World Health Organization
MUAC: Mid-upper arm circumference
WAZ: Weight-for-age *Z* score
WHZ: Weight-for-height/length *Z* score.
